# Necrotizing Pancreatitis: To Anticoagulate or Not to Anticoagulate?

**DOI:** 10.7759/cureus.23267

**Published:** 2022-03-17

**Authors:** Simon P Abi-Saleh, Ethan A Miller, Anil Magge, Mario Perez

**Affiliations:** 1 Internal Medicine, University of Connecticut School of Medicine, Farmington, USA; 2 Neurology, University of Connecticut School of Medicine, Farmington, USA; 3 Pulmonary and Critical Care Medicine, University of Connecticut School of Medicine, Farmington, USA

**Keywords:** acute necrotizing pancreatitis, systemic anticoagulation, heparin, acute pancreatitis, thrombo embolic disease, atrial fibrillation, gastro-intestinal bleed, pancreatic hemorrhage

## Abstract

Necrotizing pancreatitis is an inflammatory process that poses a strong risk of systemic venous thromboembolism. However, it is often challenging to opt for systemic anticoagulation since the disease is also associated with an increased risk of hemorrhage. Given these opposing complications, a risk versus benefit analysis has to be employed in the management of necrotizing pancreatitis on a case-by-case basis. We discuss a case where the team was faced with a dilemma regarding anticoagulation in a patient with newly developed atrial fibrillation in the setting of necrotizing pancreatitis. We found that there is a lack of guidelines that address the time of initiation and the type of systemic anticoagulation that should be administered in such patients.

## Introduction

Pancreatitis is a common gastrointestinal inflammatory process that requires urgent management and admission to a hospital. Although mortality secondary to pancreatitis has been declining, its incidence has been increasing with an annual incidence of approximately 4.9 to 40 cases per 100,000 [1]. The majority of patients present with mild pancreatitis which is commonly self-limited and resolves within one week. However, 20% of patients may develop moderate or severe pancreatitis with critical complications such as pancreatic necrosis. Along with its critical complications, there is a tremendous increase in mortality rate of 20%-40% [2].

Early diagnosis of pancreatitis is necessary for prompt treatment. The patient must meet two of the following three criteria: upper abdominal or epigastric pain, level of serum amylase or lipase more than three times the upper limit of normal, and/or positive imaging findings consistent with pancreatitis. Furthermore, it is crucial to determine the etiology of pancreatitis. The most common causes of acute pancreatitis include gallstones and alcohol abuse at 45% and 20%, respectively. Other etiologies include hypercalcemia, hypertriglyceridemia, infection, autoimmune diseases, trauma, medications, and endoscopic retrograde cholangiopancreatography (ERCP) [2].

Necrotizing pancreatitis occurs in 5%-10% of acute pancreatitis when more than 30% of the pancreas is necrosed. Acute pancreatitis can be further complicated by the collection of fluid adjacent to or within the parenchyma. These fluid collections can be further classified as sterile or infected. The mortality rate approaches 10% if it is sterile necrosis, whereas the mortality rate of infected necrosis is 20%-30% [3]. In this case presentation, we present a patient who was diagnosed with severe necrotizing pancreatitis complicated by new-onset atrial fibrillation and pulmonary embolism warranting a discussion about anticoagulation. However, prior to beginning anticoagulation, the risk of hemorrhagic pancreatitis had to be considered.

There is a paucity of literature that discusses the complications of hemorrhagic pancreatitis and further defines its risk factors and consequences. Pancreatitis can result in hemorrhage due to the inflammatory process causing erosion of major vessels ultimately leading to rupture and major bleeding. The mortality rate in hemorrhagic pancreatitis has been reported to be anywhere from 34% to 52% [4]. The issue that our case report aims to shed light on includes the ideal time and agent to initiate anticoagulation in this specific patient population without increasing the risk of fatal bleeding outcomes.

## Case presentation

A 67-year-old male with a medical history significant for coronary artery disease, essential hypertension, hyperlipidemia, and horseshoe kidney presented with epigastric pain and multiple episodes of nonbloody emesis. He reported that he first experienced abdominal pain the morning prior to presentation, which was followed by nausea and multiple episodes of vomiting. He reported drinking two to three alcoholic beverages per week. He does not have a history of cholelithiasis. He denied fever, sweats, or chills. He denied starting new medication and any recent travel to tropical areas or contact with scorpion venom.

At the emergency department, the patient was hemodynamically stable with a temperature of 36.9°C (98.4°F), a pulse of 67 beats per minute, a blood pressure of 131/87 mmHg, respiratory rate of 16 breaths per minute, and an oxygen saturation of 96% on room air. He had epigastric tenderness. His lipase was significantly elevated, greater than 6,000 U/L. Triglycerides were mildly elevated at 230 mg/dL. Aspartate aminotransferase (AST) was 210 U/L, and alanine transaminase (ALT) was 209 U/L. Total bilirubin was 1.5 mg/dL, with direct bilirubin found to be 0.9 mg/dL. His white blood cell count was 18,000/µL, and his lactic acid was 5.3 mmol/L. A computed tomography (CT) of the abdomen revealed diffuse peripancreatic edema, with no evidence of pancreatic necrosis or pseudocyst formation. The gallbladder was mildly distended with no evidence of gallbladder wall thickening, and there was no biliary duct dilation.

The patient was on medications associated with drug-induced pancreatitis including benazepril, omeprazole, atorvastatin, and prophylactic nitrofurantoin for recurrent urinary tract infections. However, these were all chronic medications that he had been taking for years. The patient was treated with lactated Ringer's infusion in the setting of pancreatitis. Magnetic resonance cholangiopancreatography (MRCP) ruled out gallstone pancreatitis. Nevertheless, the imaging revealed findings suggestive of necrotizing pancreatitis. The patient’s clinical course worsened a day after the MRCP, and he was subsequently transferred to the intensive care unit (ICU) for new-onset atrial fibrillation with rapid ventricular response and worsening respiratory status. He was found to be in distributive shock secondary to pancreatitis and was intubated and started on vasopressors.

The patient was persistently in atrial fibrillation with rapid ventricular response for the first two days in the ICU. He was ultimately started on digoxin and a beta-blocker to control his rate and was subsequently taken off vasopressors as he progressively became hemodynamically stable. He was started on heparin infusion on day 10. He was intubated for seven days for acute hypoxic respiratory failure in the setting of volume overload and bilateral pleural effusions. He also developed fever and leukocytosis. Due to the rising concern for septic shock, the patient completed a 10-day course of meropenem. His kidney function worsened, and he was diagnosed with nonoliguric acute kidney injury secondary to acute tubular necrosis in the setting of severe hypotension. He required one session of continuous renal replacement therapy (CRRT) for resistant hyperkalemia and worsening kidney function. His renal function eventually improved.

**Figure 1:**
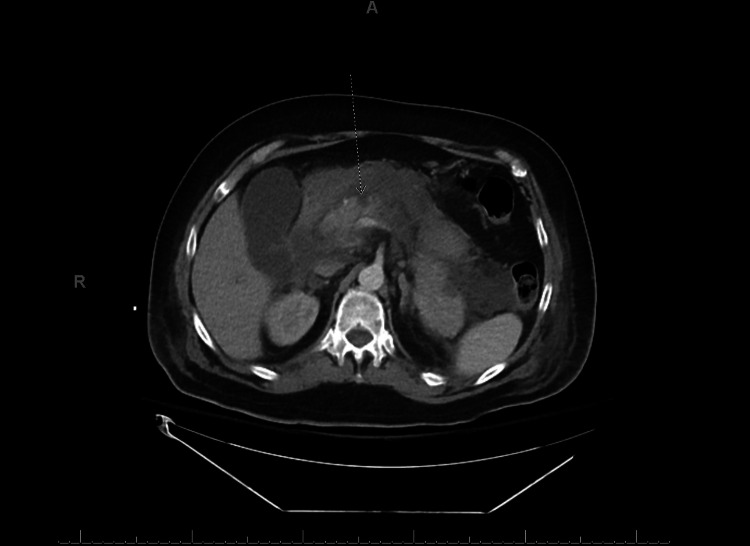
Evidence of necrotizing pancreatitis with an area of nonenhancement at the pancreatic neck indicated by the arrow. A: anterior, R: right.

**Figure 2:**
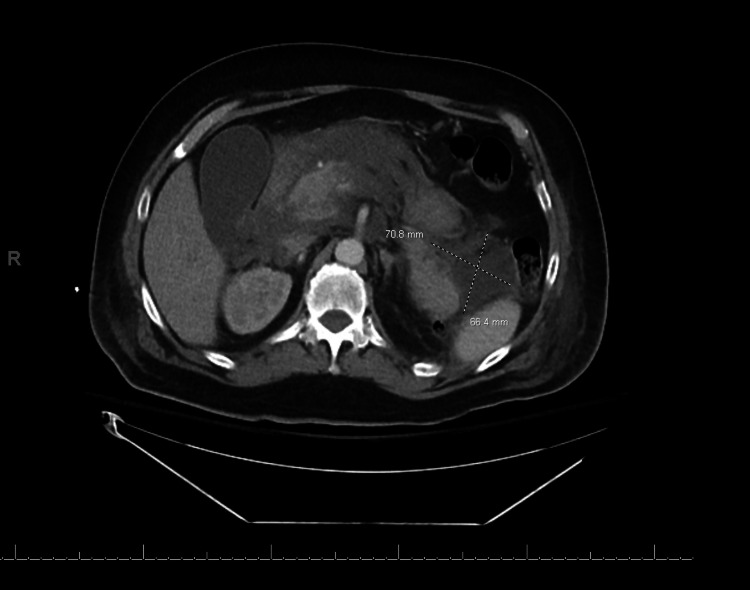
Necrotic collection in the left pararenal space measuring 7.08 x 6.6 cm. R: right.

After an 11-day stay in the ICU, the patient was transferred to an intermediate unit for further care. A CT scan of the abdomen 15 days after his admission revealed evidence of combined pancreatic (Figure 1) and peripancreatic necrosis (Figure 2). Moreover, there were findings suggestive of superior mesenteric vein (SMV) and portal vein thrombosis. In fact, the SMV appeared narrower in caliber, and the portal vein measured 9 mm in diameter, compared to 16 mm in the prior study on admission. Unfortunately, the patient was also noted to have right lower extremity thrombosis while on intravenous heparin. A CT angiography of the chest revealed pulmonary embolism in the right anterior upper lobe and middle lobe pulmonary arteries. There was evidence of elevated right heart strain, and the anticoagulation regimen was modified to systemic low molecular weight heparin (LMWH) once renal function improved.

Prior to discharge, the patient was asked to hold enalapril, nitrofurantoin, and benazepril due to association with pancreatitis with a plan to be further evaluated for pseudocyst formation by the Gastroenterology team. He was discharged on rate control and rhythm control medications for his newly diagnosed arrhythmia. He had a new diagnosis of diabetes mellitus and was discharged on appropriate antiglycemic medications. He was also started on apixaban for systemic anticoagulation in the setting of atrial fibrillation and venous thrombosis. The cause of the patient’s acute pancreatitis remained unclear. On one hand, microlithiasis or biliary sludge might have caused severe inflammation. On the other hand, drug-induced pancreatitis remained on the differential.

## Discussion

According to the 2012 Atlanta criteria, acute pancreatitis can be classified into three categories. Mild acute pancreatitis is defined as pancreatitis without organ damage such as renal or pulmonary failure and without complications such as pancreatic pseudocyst or necrosis. The other two categories include organ damage or local complications and differ in regard to the duration of the disease. Moderately severe acute pancreatitis lasts for less than 48 hours, while severe acute pancreatitis (SAP) extends beyond that time [5]. In our case, the patient was suffering from severe pancreatitis. He had parenchymal and peripancreatic necrosis, which was complicated by respiratory failure, cardiac arrhythmias, and renal failure. These complications have been associated with the proinflammatory phase of pancreatitis. Termed as a systemic inflammatory response syndrome (SIRS), phase 1 of pancreatitis occurs in the first two weeks of the disease [5]. It is at the end of this phase that a thrombotic complication occurred in our case, with clots found in the superior mesenteric vein, portal vein, and right upper and middle lobe pulmonary arteries. In fact, thromboembolism is more prevalent in necrotizing pancreatitis. In a retrospective cross-sectional study, 7.1% of patients with acute necrotizing pancreatitis had a venous thromboembolic event, whereas only 2.8% of patients with acute non-necrotizing pancreatitis had a venous thromboembolic event [6]. Moreover, patients with thromboembolism had a higher mortality rate.

Hemorrhage has been one of the most feared complications of severe pancreatitis. In a study performed by Sharma et al. [4], out of 449 patients with acute pancreatitis, 6.2% developed hemorrhage. This complication included gastrointestinal bleeds and hemorrhage into the pancreatic bed. The pathophysiology is believed to be secondary to bleeding peptic ulcers and enzymatic damage to the adjacent vascular bed such as the splenic, renal, and gastric arteries [7-8]. The mortality rate in those who suffered from bleeding complications (28.6%) was higher than that in nonbleeders (13%) [8]. Interestingly, Sharma et al. [4] noted that the median period for the onset of hemorrhage was at 26.5 days from the onset of the disease, which coincides with the second anti-inflammatory and severe immune-suppressive phase of pancreatitis [5-9].

With these two opposing complications in mind, it was difficult to decide whether the patient should be started on anticoagulation. Moreover, the fact that our patient was suffering from atrial fibrillation increased the risk of a possible thromboembolic event such as a cerebrovascular accident. In a recent retrospective study done by Zhou et al. [10], they demonstrated that early initiation of systemic anticoagulation in acute necrotizing pancreatitis decreased the event of splenic venous thrombosis by almost half. In this study, 93.5% of the study group received low molecular weight heparin in the first week of pancreatitis, and only 21% of the study group had a thrombotic splenic event. This was compared with another group of patients where only 11.5% were initiated with systemic anticoagulation during the first week of the disease, and 46% of these patients had suffered from a thrombotic event. The bleeding outcome was found to be similar in both groups; however, this outcome did not reach statistical significance. 

In our case, systemic anticoagulation was initiated with heparin 10 days after the diagnosis of acute necrotizing pancreatitis. Given this diagnosis and new-onset atrial fibrillation, our patient was at an increased risk for the development of thromboembolism. However, systemic anticoagulation was initially held due to the risk of developing hemorrhagic pancreatitis, and this might have contributed to the thromboembolic events that later developed. Although Zhou et al. [10] initiated anticoagulation sooner, and with a different agent, 21% of their patients still developed thromboembolism. Furthermore, while anticoagulation is essential in order to reduce this risk, the risk for hemorrhage must not be ignored. Sharma et al. [4] found that the risk for hemorrhagic pancreatitis still exists later in the disease course. Therefore, further research is needed to determine the ideal time and agent to initiate anticoagulation with the lowest risk of hemorrhage.

## Conclusions

The management of necrotizing pancreatitis poses many challenges. The decision for systemic anticoagulation has to be weighed against the risk of hemorrhage. Some studies have shown that the early initiation of anticoagulation has shown benefits in decreasing the occurrence of thromboembolic events. Nevertheless, more research needs to be done in order to identify the appropriate choice of anticoagulation and the ideal time to initiate anticoagulation. It is imperative to establish a clear mortality benefit for future patients while considering the risk of hemorrhage in acute necrotizing pancreatitis.
